# Endohedral metallofullerene electrides of Ca_12_O_12_ with remarkable nonlinear optical response

**DOI:** 10.1039/d0ra08571e

**Published:** 2021-01-05

**Authors:** Annum Ahsan, Saima Khan, Mazhar Amjad Gilani, Khurshid Ayub

**Affiliations:** Department of Chemistry, COMSATS University Islamabad, Abbottabad Campus Abbottabad KPK 22060 Pakistan khurshid@cuiatd.edu.pk +92-992-383591; Department of Chemistry, COMSATS University Islamabad, Lahore Campus Defence Road, Off-Raiwind Road Lahore 54000 Pakistan mazhargilani@cuilahore.edu.pk

## Abstract

Herein, the structural, electronic, thermodynamic, linear and nonlinear optical properties of inorganic electrides, generated by alkali metal doping in group II–VI Ca_12_O_12_ fullerene, are studied. Endohedral doping of alkali metal leads to the formation of electrides whereas no such phenomenon is seen for exohedral doping. The electride nature of the endohedral fullerenes is confirmed through the analysis of frontier molecular orbitals. The results show that doping of alkali metal atoms leads to a reduction of the HOMO–LUMO gap and increase of the dipole moment, polarizability and hyperpolarizability of nanocages. Doping causes shifting of electrons from alkali metal atoms towards the Ca_12_O_12_ nanocage, which serve as excess electrons. Furthermore, the participation of excess electrons for enhancing the NLO response of these nanocages has been confirmed through the calculation of hyperpolarizability (*β*_o_). For exploring the controlling factors of hyperpolarizability, a two level model has been employed and the direct relation of hyperpolarizability with Δ*μ* & *f*_o_, while an inverse relation of hyperpolarizability with Δ*E* has been studied. The electrides possess remarkable nonlinear response where the highest hyperpolarizability can reach up to 1.0 × 10^6^ a.u. for *endo*-K@Ca_12_O_12_. This electride has the lowest Δ*E* of 0.63 eV among all compounds studied here. These intriguing results will be expedient for promoting the potential applications of the Ca_12_O_12_-based nano systems in high-performance nonlinear optical (NLO) materials.

## Abstract 2

Electride formation by alkali metal doping with a drastic increase in hyperpolarizability

## Graphical abstract

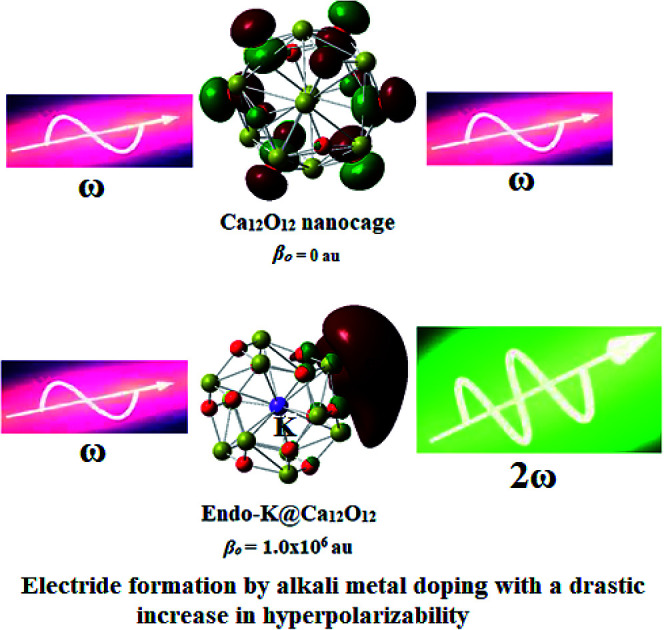

## 1. Introduction

Since the discovery of (C_60_) fullerene in 1985 by Kroto *et al.*^1^ extensive research on fullerenes revealed that the spherical allotropes of carbon possess very interesting properties. Because of the revelation of their exclusive properties and diverse applications, research has been extensively dedicated to explore new nanoscale materials.^2–5^ Recently, a variety of different inorganic-based fullerene like nanomaterials have been reported.^6,7^ Among these inorganic nanocages, very appealing nanocages are the ones with general formula (XY)_*n*_, where *n* is the number of atoms. Among these nanocages, the most stable nanocages are the ones with *n* = 12.^8–11^ However, adequate explanation about this magic number *i.e.* 12 is not reported but one fact that favors the fascinating nature of these (XY)_12_ nanocages is that these cages satisfy the tetragonal rule.^12^ These (XY)_12_ nanoclusters are condensed octahedrons comprising of six squares and eight hexagons. Among these cages, B_12_N_12_ and Al_12_N_12_ are very well-known nanostructures.^13,14^ These nanocages besides various other applications are potential candidates for smart materials with large nonlinear optical response.

Nonlinear optical materials have received great attention due to wide range of applications^15^ such as optical communication,^16^ optical computing,^17,18^ dynamic image processing and other laser devices.^19^ In this regard, different strategies for designing NLO materials have also been introduced. One of the strategies *i.e.* introduction of excess electron^20–29^ into different structures is employed recently, where a metal atom (preferably alkali metal atom) is doped into different structures (mostly the cages). The presence of excess electron enhances the nonlinear optical properties of the structures, especially their hyperpolarizability (*β*_o_). Excess electrons can be introduced in a system by doping with alkali metals,^27,30^ superalkalis,^31,32^ transition metals^33,34^ and alkaline earth metals.^35,36^ The literature reveals extensive examples where transition metals or alkali metal atoms are introduced in the system by exohedral,^33,37–39^ endohedral^40,41^ and substitutional doping.^29,42–44^ Two main classes of compounds which contain free excess electrons are electrides and alkalides. They possess significantly higher nonlinear optical responses.^23,25,26,45^ Remarkable NLO response of electrides and alkalides has led the researchers to design new electrides and alkalides with even better properties. For this purpose, alkali metal/superalkali doped complexes of 2^6^ Adz, 3^6^ Adz,^46^ calix^4^ pyrrole,^24^ cyclic polyamine,^47^ cyclacene,^48^ organic amines,^49,50^ fluorocarbon^51^*etc.* are reported. Electrides are formed when ns^1^ electron of the alkali metal atom is pushed out by the complexant which then becomes an excess electron in the system. These well-known (X_12_Y_12_) nanocages are studied for their nonlinear optical response through doping of metals. For example, Huang *et al.* have shown through density functional calculations that alkali metal atoms doped Al_12_N_12_ nanocages show remarkable nonlinear optical response (because of introduction of excess electron) with the highest hyperpolarizability of 8.89 × 10^5^ a.u. for Li@r_6_–Al_12_N_12_.^13^ Exohedral as well as endohedral doping of alkali metals on different organic^52^/inorganic fullerenes such as B_12_P_12_, Al_12_P_12_,^38^ B_12_N_12_ (ref. ^53^) and other related structures consistently reveal that alkali metals doping is an effective strategy for enhancing the nonlinear optical response through the introduction of excess electrons.

Density functional calculations for nonlinear optical properties of alkali metal substituted boron nitride (MB_12_N_11_/MB_11_N_12_) nanocages reveal that the substitutional doping is also an effective strategy where the first hyperpolarizability (*β*_o_) of B_12_N_12_ is increased up to 1.3 × 10^4^ a.u. for KB_12_N_11_ nanocage (the *β*_o_ was 0 a.u. for pure B_12_N_12_).^29^ Quite similar to alkali metal doping, superalkali doping also causes significant enhancement in the first hyperpolarizability.^54,55^ Computational results reveal that Li_3_O@Al_12_N_12_ contains diffuse excess electrons with considerable first hyperpolarizability (*β*_o_) up to 1.86 × 10^7^ a.u.^11^

Other than these clusters, metal oxide clusters of X_12_Y_12_ type *i.e.* Be_12_O_12_, Mg_12_O_12_, Ca_12_O_12_, Zn_12_O_12_*etc.* are studied frequently as they show remarkable properties because of the larger ionic character of bonds in these metal-oxides. These metal oxide clusters can be used for catalysis,^56^ adsorption processes,^57,58^ gas sensors^59^ and nonlinear optical applications as well (where NLO properties are induced through doping). The studies involving nonlinear optical properties of such metal oxide clusters include doping of alkali metals, superalkali clusters and transition metals in Be_12_O_12_ and Mg_12_O_12_ nanoclusters. The interaction of alkali metals (Li, Na and K) with Be_12_O_12_ and Mg_12_O_12_ nanocages introduces the excess electron into them which results in the reduction of their HOMO–LUMO gaps and enhancement of their first hyperpolarizabilities from 0.091 and 0.081 a.u. to 9.4 × 10^3^ and 2.3 × 10^4^ a.u. for Be_12_O_12_ and Mg_12_O_12_ nanocages, respectively.^60^ Similarly, the adsorption of alkali metal oxides M_*n*_O (M = Li, Na and K; *n* = 2, 3 and 4) on Mg_12_O_12_ nanocages also shows reduction in HOMO–LUMO gap along with a significant increase in hyperpolarizability. In this case, the highest increase is seen in the case of superalkalis (M_3_O, where M = Li, Na and K) on Mg_12_O_12_ with the highest hyperpolarizability of 6.0 × 10^6^ a.u. for K_3_O@Mg_12_O_12_.^61^ Moreover, substitutional doping of transition metals on Mg_12_O_12_ nanocages was studied where doping of transition metals significantly increases the hyperpolarizability of these nanocages and the highest hyperpolarizability of 4.7 × 10^4^ a.u. is observed for Sc@Mg_12_O_12_.^62^ In a similar way, NLO properties of superalkalis (Li_3_O, Na_3_O and K_3_O) doped Zn_12_O_12_ clusters are studied.^63^ These superalkalis doped nanocages also possess remarkably high first hyperpolarizability (*β*_o_) values up to 3.9 × 10^5^ a.u. for K_3_O@Zn_12_O_12_.

Among Be_12_O_12_, Mg_12_O_12_ and Ca_12_O_12_ nanoclusters, Mg_12_O_12_ has been studied extensively for its physical and chemical properties as compared to Be_12_O_12_ and Ca_12_O_12_. The interest in such a nanocage was developed due to ionic character of Mg–O bond in Mg_12_O_12_ nanocluster. The ionic character provides this cluster remarkable properties and invokes the need for its further investigation. Other than Mg_12_O_12_, Ca_12_O_12_ nanocage with the larger cavity size and larger ionic radius^12^ (possessing the appealing properties) is more interesting candidate for further studies especially for its nonlinear optical properties. Ca_12_O_12_ is also expected to show NLO response when doped with metals. In this report, we are dedicated to study the doping of Ca_12_O_12_ with alkali metals both exohedrally and endohedrally. The effect of doping on different sites of this nanocage on NLO properties is investigated in detail.

## 2. Computational methodology

Geometry optimization of M@Ca_12_O_12_ (M = Li, Na and K) nanoclusters is performed at ωB97X-D/6-31G(d,p) level of theory. ωB97X-D is a reliable method for the geometry optimization of systems containing alkali metals and systems with non-covalent interactions.^64–67^ Because of the presence of both these features in our system, this functional is selected in combination with 6-31G(d,p) basis set. ωB97X-D is a long range and dispersion corrected method^64,66^ which is well known for the prediction of non-covalent interactions. For optimization, a number of possible orientations for alkali metals on calcium oxide nanocages are considered. Then, frequency analysis is performed for all these structures at the same level of theory to confirm that the optimized structures correspond to real local minima (absence of imaginary frequencies). The stabilities of these alkali metals doped calcium oxide nanocages are evaluated by the calculation of interaction energies at the same level of theory through the formula *i.e.*

1 *E*_int_ = [*E*(M@Ca_12_O_12_)] − [*E*(M) + *E*(Ca_12_O_12_)] (where, M = Li, Na and K)

where *E*(Ca_12_O_12_), *E*(M) and *E*(M@Ca_12_O_12_) are the total energies of the undoped Ca_12_O_12_ nanocage, corresponding alkali metal unit (Li, Na and K) and doped system, respectively. For the exploration of electronic properties, HOMO–LUMO gap (*E*_H–L_) values are calculated and natural bond orbitals (NBO) are analyzed. *E*_H–L_ is calculated by using the formula; *E*_H–L_ = *E*_H_ − *E*_L_, where *E*_H_ and *E*_L_ are the energies of highest occupied molecular orbitals and lowest unoccupied molecular orbitals, respectively. The partial density of states (PDOS) of all the structures are plotted by using Multiwfn software for graphical representation of electronic properties.^68^ Moreover, other properties such as vertical ionization energies (VIE), dipole moments, ultraviolet-visible-infrared (UV-VIS-NIR) absorption spectra, polarizability (*α*_o_) and first hyperpolarizability (*β*_o_) are also calculated at ωB97X-D/6-31G(d,p) level of theory.

For the calculation of first hyperpolarizability (*β*_o_), the crucial selection of a functional is required. Range separation is very important for accurate calculations of hyperpolarizabilities. Earlier studies^69,70^ have reported that for better estimates of nonlinear optical properties, a full range-separated functional is preferable. ωB97X-D is reliable in this regard as well.

The mean polarizability (*α*_o_) and hyperpolarizability (*β*_o_) are defined as follows:

2 *α*_o_ = 1/3(*α*_*xx*_ + *α*_*yy*_ + *α*_*zz*_)

3 *β*_o_ = [*β*_*x*_^2^ + *β*_*y*_^2^ + *β*_*z*_^2^]^1/2^

where *β*_*x*_ = *β*_*xxx*_ + *β*_*xyy*_ + *β*_*xzz*_, *β*_*y*_ = *β*_*yyy*_ + *β*_*yzz*_ + *β*_*yxx*_ and *β*_*z*_ = *β*_*zzz*_ + *β*_*zxx*_ + *β*_*zyy*_.

The time-dependent density functional theory (TD-DFT) calculations are performed at the TD-ωB97X-D/6-31G(d,p) level to get the crucial excitation energies (Δ*E*) and oscillator strength (*f*_o_). All calculations are carried out by using the Gaussian 09 program package.^71^ Molecular structures and orbitals are generated with the Gauss View program.

## 3. Results and discussions

### 3.1. Geometrical characteristics

**Fig. 1:**
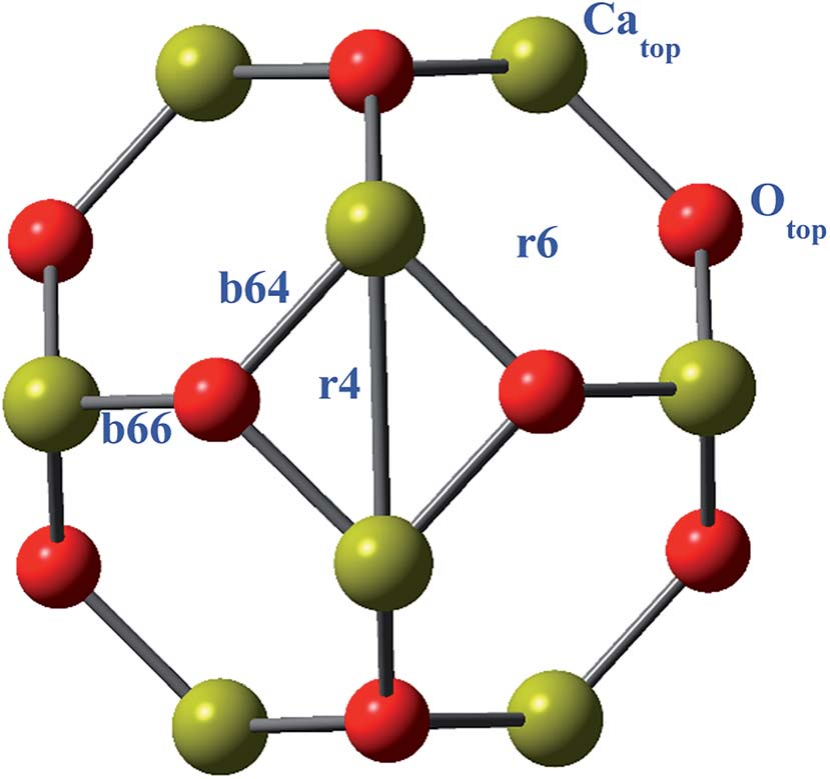
Illustration of different orientations on Ca_12_O_12_ nanocage.

Geometries of the alkali metals (Li, Na and K) doped (exohedrally and endohedrally) Ca_12_O_12_ nanocages are studied at ωB97X-D/6-31G(d,p) level of theory. The nanocage consists of six hexagonal rings and four tetrahedral rings. For doping alkali metals exohedrally, six different sites are selected namely, *b*_64_, *b*_66_, *r*_6_, *r*_4_, Ca_top_ and O_top_ (Fig. 1). The site “*b*” represents the cases where alkali metal resides on a bond whereas “*r*” represents the cases where alkali metal is located on a ring. Specifically, *b*_66_ represents the case where alkali metal is doped on Ca–O bond shared between two six membered rings of the cage while *b*_64_ represents the case where alkali metal is present on Ca–O bond shared between a six membered ring and a four membered ring of the cage. Similarly, *r*_4_ and *r*_6_ represent the cases where alkali metal is placed at the center of four and six membered ring of the cage, respectively. However, Ca_top_ and O_top_ represent the cases where alkali metal resides on the top of Ca and O atom of the cage, respectively. For endohedral doping, alkali metals are placed inside the cage.

**Fig. 2:**
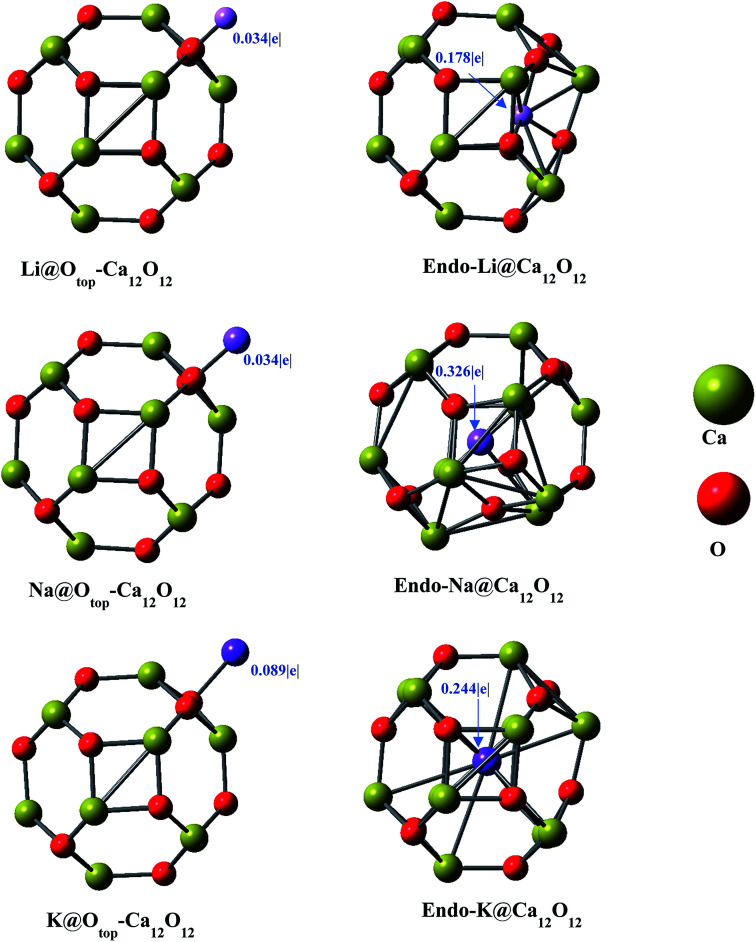
Optimized geometries of M@Ca_12_O_12_ (M = Li, Na and K) complexes.

The Li doped (at different positions) nanocages are optimized in *C*_1_ symmetry. For different input geometries of lithium atom on cage (*b*_66_, *b*_64_, *r*_4_, *r*_6_, Ca_top_ and O_top_), the optimized geometries are O_top_ except for *r*_6_ input where the optimized geometry matches with the input geometry (Fig. 2). This is because of oxygen's electronegative nature for which the electropositive metal is driven to lie on top of it. However, when doped at the center of six membered ring exohedrally (*r*_6_ position), Li moves inward towards inside of the cage because of the size of hexagon larger enough to allow the smaller sized metal (Li) to move in, and hence optimized geometry contains Li inside the cage (not at the center of cage but at one side inside the cage) (Fig. 2). Moreover, upon endohedral doping Li moves to one side inside the cage because of its smaller size not fitting center of the cavity. This geometry is similar to the optimized geometry obtained as a result of doping Li at *r*_6_ position.

Similarly, the same positions for optimization are selected for Na with respect of the Ca_12_O_12_ nanocage. Na optimizes at O_top_ (because of its higher electronegativity) for all inputs including *r*_6_ site (unlike Li) which may be attributed to its larger size. The larger size of sodium does not allow it to cross the six membered ring. Moreover, endohedrally doped Na optimizes exactly at the center of the cage (with some distortion of the cage because of its larger size) because of its size fitting the cavity (Fig. 2). In the similar way, the same positions are selected for K and the optimized geometries show that the results are similar to that of the sodium doping. Exohedrally doped K optimizes at oxygen top finally and endohedrally doped K optimizes exactly at the center of the cage because of its size fitting the cavity (with some distortion of the cage as well) (Fig. 2). For all alkali metal atoms, only two structures could be identified; endohedral doped alkali metal in Ca_12_O_12_ and O_top_ Ca_12_O_12_.

Concerning the thermodynamic stability of alkali metals doped Ca_12_O_12_ complexes, interaction energies are calculated by using formula given as eqn (1). All the optimized geometries (both with exohedrally doped and endohedrally doped alkali metals) show negative values of interaction energies which reveal the stability of these complexes. The interaction energy of Li@O_top_–Ca_12_O_12_ is −68.88 kcal mol^−1^ while for *endo*-Li@Ca_12_O_12_ (optimized geometry with Li lying endohedrally), the interaction energy is −77.47 kcal mol^−1^. The interaction energy for endohedrally doped Li complex is higher than that of Li@O_top_–Ca_12_O_12_. For Na doped complexes, the interaction energies for Na@O_top_–Ca_12_O_12_ and *endo*-Na@Ca_12_O_12_ are −52.78 kcal mol^−1^ and −59.81 kcal mol^−1^, respectively. In this case, the higher value of interaction energy is also for endohedral doping which reflects its higher stability compared to the exohedral doping. Moreover, the interaction energies are higher for lithium complexes than those of sodium complexes which may be attributed to higher charge density in the former than the latter. For K doped complexes, the interaction energies are −45.31 kcal mol^−1^ and −47.87 kcal mol^−1^ for K@O_top_–Ca_12_O_12_ and *endo*-K@C_12_O_12,_ respectively. Quite similar to the lithium and sodium complexes, the higher value of interaction energy is seen for endohedral doping of K. Overall, the interaction energies for Li@Ca_12_O_12_ are higher as compared to the interaction energies calculated for Na@Ca_12_O_12_. While the interaction energies for Na@Ca_12_O_12_ are higher than interaction energies calculated for K@Ca_12_O_12_ complexes. This shows the better interaction of smaller sized metal with the cage as compared to the larger sized metal atoms. The interaction becomes weaker with the increase in size of metal atom. This is consistent with various reports in the literature where higher interaction energies are observed with smaller alkali metals.^60^

#### Dipole moment

The dipole moment is defined as the product of charges and distance between them. The higher the point charges, the more the dipole moment. Similarly, the more the interaction distance between the charges, the more the dipole moment. For Li doped Ca_12_O_12_ nanocages, the dipole moments for Li@O_top_–Ca_12_O_12_ and *endo*-Li@Ca_12_O_12_ are 5.43 and 4.47 D, respectively. The dipole moment of Li@O_top_–Ca_12_O_12_ geometry is higher than that of *endo*-Li@Ca_12_O_12_ which may be attributed to higher separation between point charges, although the point charges are higher in the *endo*-Li@Ca_12_O_12_. This reveals that the separation between charges is the deciding factor for dipole moment.

For Na doped at different positions, dipole moments for Na@O_top_–Ca_12_O_12_ and *endo*-Na@Ca_12_O_12_ are 4.77 D and 8.17 D, respectively. The larger charge transfer from internally lying Na to the cage might be the reason for this larger dipole moment for endohedrally lying Na. For K doped at different positions, the dipole moments for K@O_top_–Ca_12_O_12_ and *endo*-K@Ca_12_O_12_ are 3.45 D and 10.94 D, respectively. The higher value (10.94 D) is because of the larger interaction distance between the charges in this case, as compared to doping at other sites. In this case, the reason might be the greater charge transfer from K to the cage as compared to K doped at other sites. For all these geometries, the direction of dipole moment is away from the alkali metal atoms (towards the cage) indicating that the charge is transferred from the electropositive metal to the electronegative oxygen atoms of the nanocage.

Overall, the dipole moments calculated for exohedrally doped metal complexes show that the values of *μ*_o_ are higher for Li@Ca_12_O_12_ complexes as compared to the Na@Ca_12_O_12_ which in turn are higher than the K@Ca_12_O_12_ complexes. For endohedral doping, the larger metals show larger values *i.e.* 8.17 D and 10.94 D for dipole moment (for Na and K, respectively) which is due to the greater transfer of charge as compared to the charge transfer in case of Li doped endohedrally. This is because of the large volume size of the Ca_12_O_12_ encapsulating the larger ions in a better way resulting into more charge transfer as compared to the cases where there are the smaller ions.

### 3.2. Electronic properties

The electronic properties *i.e.* NBO charges and HOMO–LUMO gaps are studied. The results of the NBO analyses (Table 1) show positive charges on alkali metals with negative charges on oxygen atoms. An increase in magnitude of negative charge on O in doped nanocages as compared to the bare nanocage is observed, which indicates that the charge has been transferred from alkali metal toward the cage. The cases, where Li lies outside the nanocage (O_top_ position, Li@O_top_–Ca_12_O_12_), the NBO charge of 0.034|*e*| on Li is observed. However, the NBO charge on Li is 0.178|*e*| in *endo*-Li@Ca_12_O_12_. The higher value of positive charge on Li in *endo*-Li@Ca_12_O_12_ is because of its interaction with larger number of oxygen atoms. In *endo*-Li@Ca_12_O_12_, Li is surrounded by three oxygen atoms (Fig. 2), whereas Li interacts with single oxygen in Li@O_top_–Ca_12_O_12_. The increase in number of interacting oxygen atoms increases the charge transfer from metal toward atoms of the cage.

**Table 1:** Symmetries (Sym.), NBO charges on metal atoms (*Q*_M_^+^, in |*e*|), interaction energies (*E*_int_, in kcal mol^−1^), ground state dipole moments (*μ*_o_, in Debye), Vertical Ionization Potentials (VIP, in eV), energies of HOMO and LUMO (*E*_HOMO_ and *E*_LUMO_, in eV) and HOMO–LUMO Gaps (H–L gaps, in eV) in the M@Ca_12_O_12_ (M = Li, Na and K) compounds

M@Ca_12_O_12_	Sym.	*Q*_M_^+^	*E*_int_	*μ*_o_	VIP	*E*_HOMO_	*E*_LUMO_	H–L gap
*endo*-Li@Ca_12_O_12_	*C*_1_	0.178	−77.47	4.47	3.09	−3.09	0.08	3.01
Li@O_top_–Ca_12_O_12_	*C*_1_	0.034	−68.88	5.43	3.94	−3.94	0.17	3.77
*endo*-Na@Ca_12_O_12_	*C*_1_	0.326	−59.81	8.17	3.24	−3.24	0.03	3.21
Na@O_top_–Ca_12_O_12_	*C*_1_	0.034	−52.77	4.77	3.92	−3.92	0.18	3.74
*endo*-K@Ca_12_O_12_	*C*_1_	0.244	−47.87	10.94	2.63	−2.63	0.05	2.58
K@O_top_–Ca_12_O_12_	*C*_1_	0.089	−45.31	3.45	3.57	−3.57	0.16	3.41

In case of Na doped Ca_12_O_12_ nanocages, the NBO charge of 0.034|*e*| is observed for Na@O_top_–Ca_12_O_12_. However, the NBO charge is 0.326|*e*| in *endo*-Na@Ca_12_O_12_. The reasons for higher charge on Na in *endo*-Na@Ca_12_O_12_ are very similar to those for *endo*-Li@Ca_12_O_12_. However, the charge on Na in *endo*-Na@Ca_12_O_12_ is higher (than that of lithium in *endo*-Li@Ca_12_O_12_) because it is surrounded by larger number of atoms, as compared to Li which is surrounded by three atoms in *endo*-Li@Ca_12_O_12._ The NBO analysis of the K doped Ca_12_O_12_ nanocages shows the NBO charge of 0.089|*e*| on K in K@O_top_–Ca_12_O_12_. The NBO charge of K in *endo*-K@Ca_12_O_12_ is 0.244|*e*|. This is because of the fact that K is surrounded by large number of electronegative oxygen atoms which enhances the transfer of charge. Comparing *endo*-M@Ca_12_O_12_ nanocages, the charge on Na (0.326|*e*|) is larger compared to Li (0.178|*e*|) and K (0.244|*e*|). This may be due to slight distortion of cage in the presence of Na. Distortion occurs in such a way that it causes some of the atoms of the cage to come closer to Na, thereby enhancing the charge transfer. Such a distortion is not observed in case of K because of its large size which hardly fits in the cavity. The potassium atom lies at the center of the cage (almost equidistant from all the surrounding atoms). On the other hand, Li in *endo*-Li@Ca_12_O_12_ does not stay at the center of the cage rather it is shifted to one side (inside the cage) without causing any distortion of the cage.

**Fig. 3:**
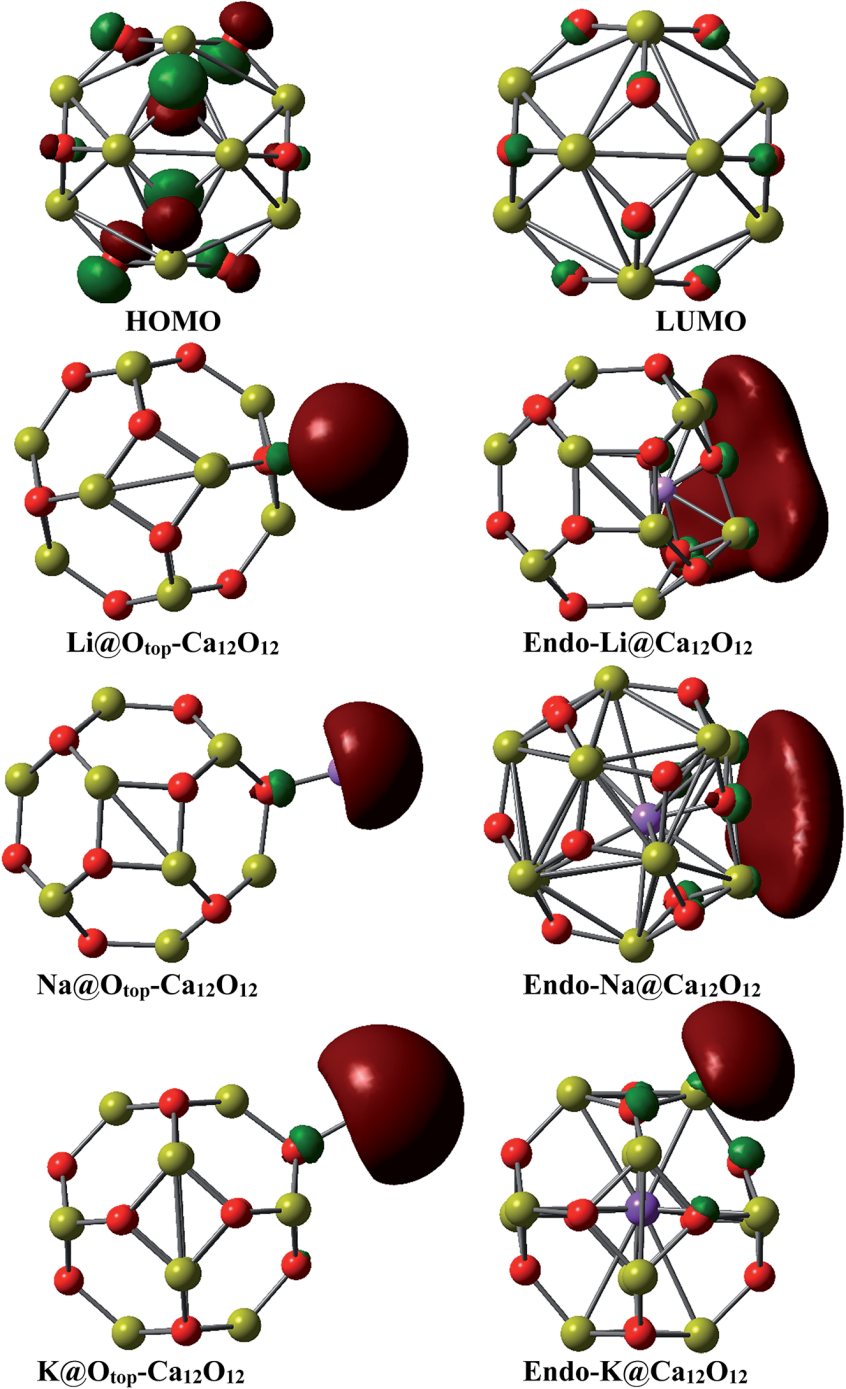
HOMOs of M@Ca_12_O_12_ nanocages where M = Li, Na and K.

The pictorial representation of HOMO and LUMO orbitals is given in Fig. 3. The frontier molecular orbital analysis for the pure cage depicts that the HOMO is concentrated on O atoms of the cage. For the doped nanocages, the HOMO–LUMO diagrams depict the distribution of the densities is changed. In case of exohedral doping, HOMO lies on the dopant *i.e.* alkali metal atom. The position of HOMO reveals these materials are excess electron compounds. In case of endohedral doping, HOMO lies outside the cage. Careful analysis of the distribution of densities in the endohedral complexes reveals that these are electrides where densities of HOMO are present in empty spaces. These excess electrons don't belong to any atom rather they are present in empty spaces. The behavior of these endohedral complexes is quite contrary to the endohedral complexes based on group III–V fullerenes which we had reported previously. In endohedral complexes of alkali metals in B_12_N_12_, B_12_P_12_, Al_12_N_12_ and Al_12_P_12_, no such electride behavior was seen.

There is a slight decrease in the HOMO–LUMO gap as well (Table 1). The reason for the decrease in the *E*_H–L_ gap is the increase in the energies of HOMOs and decrease in the energies of LUMOs after doping. The energies of HOMOs are increased due to the presence of excess electrons. The bare nanocage shows the H–L gap of 3.85 eV with *E*_HOMO_ = −6.77 eV and *E*_LUMO_ = 2.93 eV. HOMO–LUMO gap analysis reveals that the cases where metal lies endohedrally (*endo*-Li@Ca_12_O_12_, *endo*-Na@Ca_12_O_12_ and *endo*-K@Ca_12_O_12_), the gaps are lower than their respective M@*exo*-Ca_12_O_12_ cases. For example, the H–L gap of *endo*-Li@Ca_12_O_12_ (3.01 eV) is lower than Li@*exo*-Ca_12_O_12_ (3.77 eV), the H–L gap of *endo*-Na@Ca_12_O_12_ (3.21 eV) is lower than Na@*exo*-Ca_12_O_12_ (3.74 eV) and the H–L gap of *endo*-K@Ca_12_O_12_ (2.58 eV) is lower than K@*exo*-@Ca_12_O_12_ (3.41 eV). The lower HOMO–LUMO gaps for endohedral complexes are due to the electride characteristic of endohedral complexes. Such electrides are well known for low HOMO–LUMO gaps. Although the HOMO–LUMO gaps for all these geometries are lower but yet, these gaps are moderately high to impart enough electronic stability.

Vertical ionization potential (VIP) of these excess electron compounds is comparatively higher than the other excess electron compounds in literature which shows the electronic stability in these NLO compounds. However, VIP in all these compounds also depends upon the position of doping. For endohedrally doped metal complexes, VIP is lower as compared to the exohedrally doped metal complexes.

#### Density of states

**Fig. 4:**
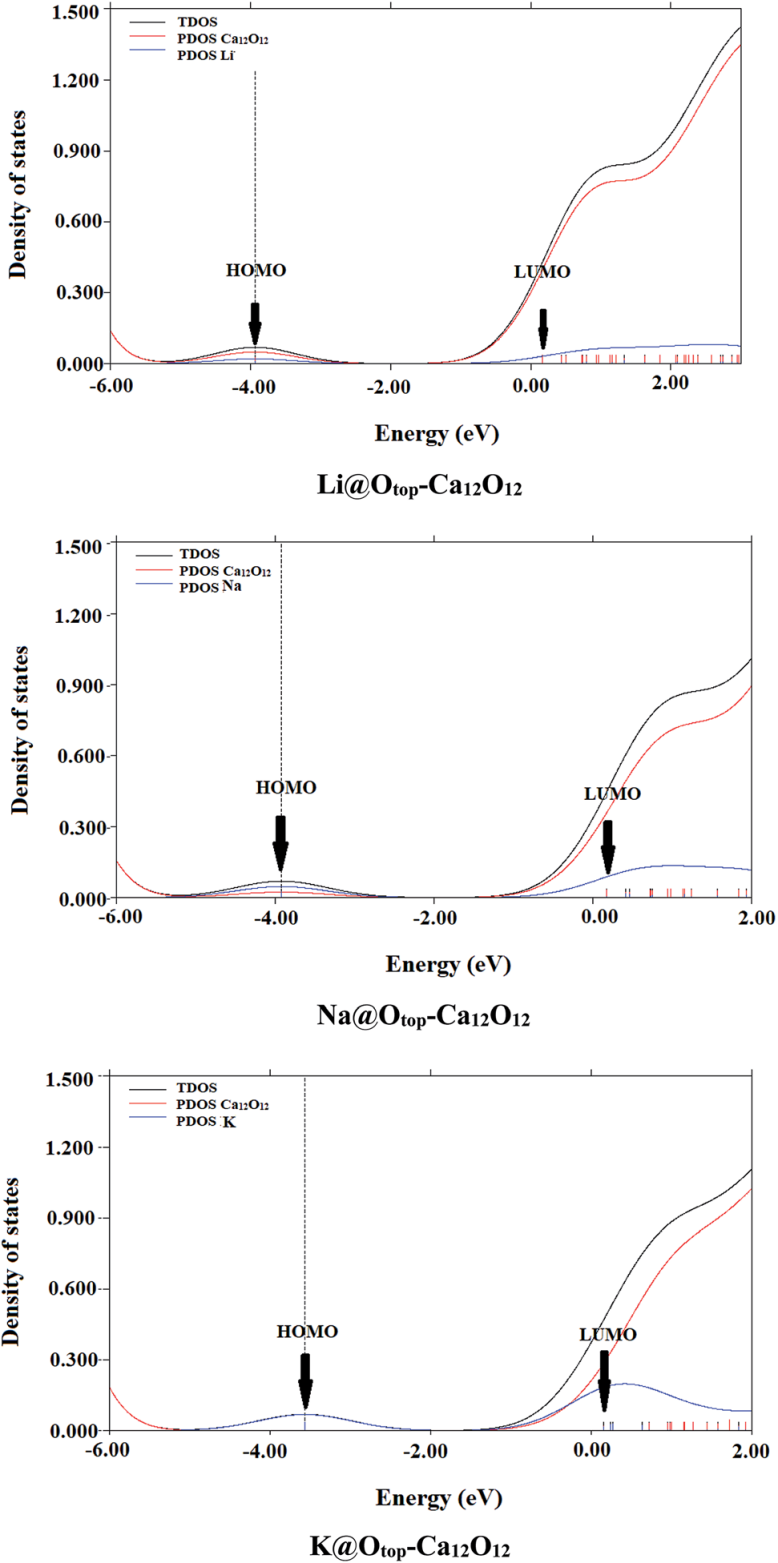
PDOS spectral analysis of M@O_top_–C_12_O_12_ (M = Li, Na and K).

**Fig. 5:**
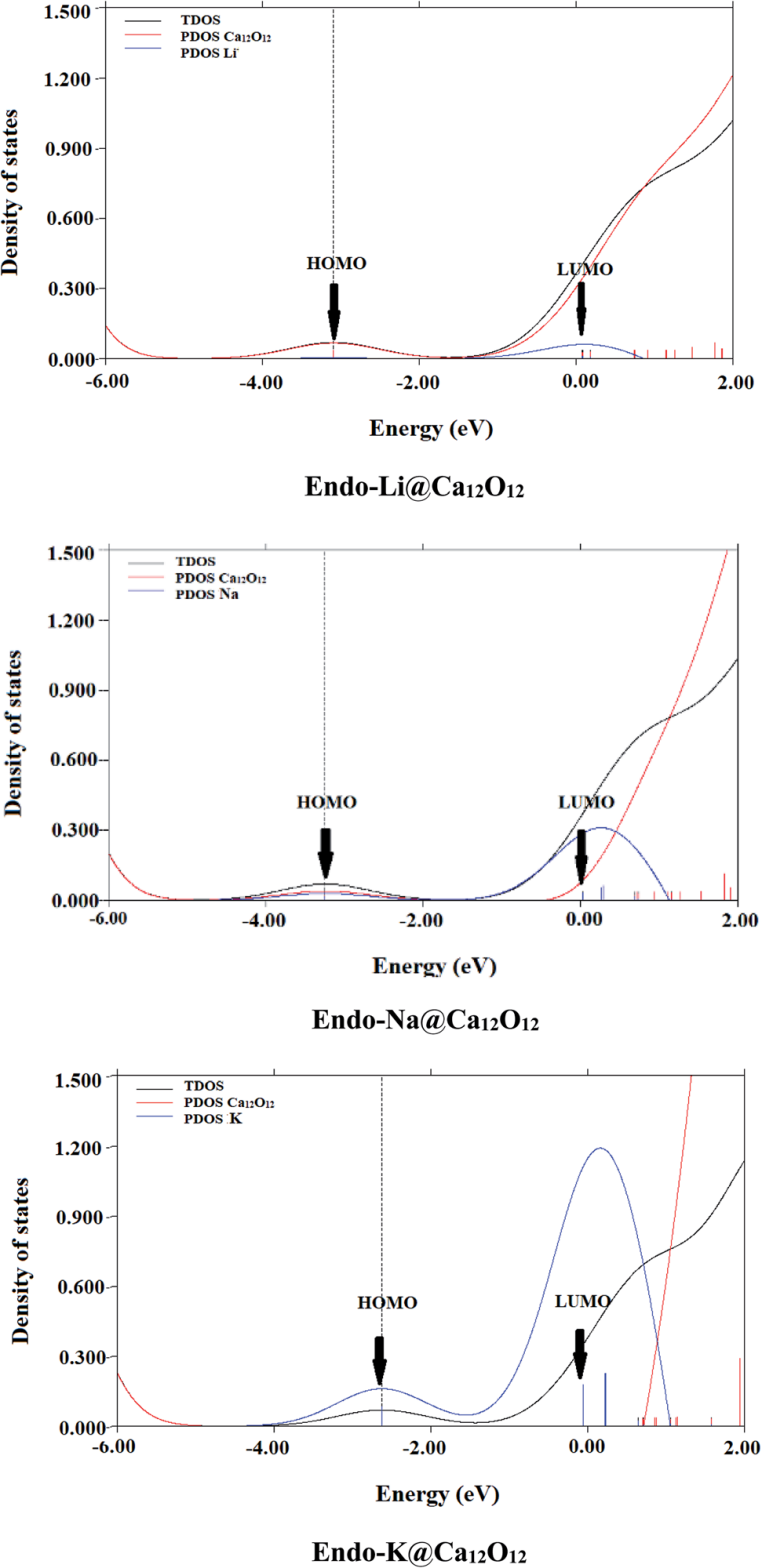
PDOS spectral analysis of *endo*-M@C_12_O_12_ (M = Li, Na and K).

For further confirmation of the electronic behavior of these alkali metals doped Ca_12_O_12_ nanocages, partial density of states (PDOS) analyses are performed. TDOS spectrum of the pure nanocage and the PDOS spectra of doped nanocages are generated and are given in Fig. 4 and 5. All these PDOS spectra indicate the contribution of alkali metal towards HOMOs.

The comparison of spectrum of bare nanocage and spectra of doped nanocages clearly show the reduction in *E*_H–L_ gap of doped cages compared to the bare nanocage which is attributed to excess electrons generated by the alkali metals. Doping alkali metals on Ca_12_O_12_ nanocage creates new energy levels, as a result of the transfer of excess electrons from alkali metals to the nanocage. The newly generated HOMOs have higher energy compared to the energies of HOMOs of pure nanocage.

#### Absorption analysis

For frequency doubling in second harmonic generation, high performance NLO materials are used. Therefore, the transparency of the NLO materials toward the laser light (which is utilized) is very important. For the investigation of transparency of the designed electrides and excess electron compounds, UV-VIS-NIR absorption analysis is performed and *λ*_max_ for the studied compounds are given in Table 2. The main absorption regions of all isomers lie in VIS-NIR. It is observed that *λ*_max_ of all of the three endohedrally doped metal geometries are higher than their respective exohedrally doped geometries. *endo*-Li@Ca_12_O_12_ shows *λ*_max_ of 2037 nm, significantly higher than its respective Li@O_top_–Ca_12_O_12_ geometry (*λ*_max_ of 689 nm). Similarly, *endo*-Na@Ca_12_O_12_ and *endo*-K@Ca_12_O_12_ geometries show *λ*_max_ of 1048 and 1752 nm, higher than the *λ*_max_ of Na@O_top_–Ca_12_O_12_ and K@O_top_–Ca_12_O_12_*i.e.* 755 and 1064 nm, respectively. Comparing absorption wavelength with *E*_H–L_, it is observed that *endo*-geometries with higher *λ*_max_ values possess lower *E*_H–L_ values as compared to their respective *exo*-geometries which possess lower *λ*_max_ but higher *E*_H–L_

**Table 2:** Polarizability (*α*_o_, in a.u.), hyperpolarizibility (*β*_o_, in a.u.), wavelength (*λ*_max_, in nm), oscillator strength (*f*_o_, in a.u.), transition energy (Δ*E*, in eV), differences in dipole moments (Δ*μ*, in D) between the ground and excited states of the crucial excited states and dominated transitions in the M@Ca_12_O_12_ (M = Li, Na and K) compounds

M@Ca_12_O_12_	*α*_o_	*β*_o_	*λ*_max_	*f*_o_	Δ*E*	Δ*μ*	Dominated transitions
*endo*-Li@Ca_12_O_12_	1373.72	1.5 × 10^5^	2037	0.2290	0.6086	0.02	H → L
Li@O_top_–Ca_12_O_12_	582.46	2.4 × 10^4^	689	0.2243	1.7987	1.46	H → L
*endo*-Na@Ca_12_O_12_	622.85	3.5 × 10^4^	1048	0.2203	1.1827	0.02	H → L+1
Na@O_top_–Ca_12_O_12_	662.02	1.6 × 10^4^	755	0.3407	1.6402	3.34	H → L
*endo*-K@Ca_12_O_12_	1427.86	1.0 × 10^6^	1752	0.2566	0.7074	0.97	H → L+2
K@O_top_–Ca_12_O_12_	1061.59	1.2 × 10^4^	1064	0.3526	1.1643	1.39	H → L

### 3.3. Nonlinear optical properties

It is reported previously in the literature that NLO response of a system can be enhanced by introducing the excess electrons into it. The excess electrons in the system result in increasing the energy of HOMO which in turn reduce the *E*_H–L_ and increase the first hyperpolarizability (*β*_o_) value of the system. In these metal doped Ca_12_O_12_ nanocages, the presence of excess electron (which has been confirmed through the charge analysis and HOMO analysis) awards large NLO response to the system. First hyperpolarizability (*β*_o_) is a decisive factor for NLO response of any system. In this regard, polarizability (*α*_o_) and hyperpolarizability (*β*_o_) are calculated by using eqn (2) and (3) and are given in Table 2.

Isolated Ca_12_O_12_ nanocage is centrosymmetric, therefore first hyperpolarizability (*β*_o_) and dipole moment (*μ*_o_) of pure nanocage are zero. The doping of alkali metals brings significant enhancement of the dipole moment (*μ*_o_) and first hyperpolarizability (*β*_o_) for the Ca_12_O_12_ nanocage. This is due to the distortion of symmetry of the nanocage. The calculated values of polarizability show an increase with the increase in size of doped alkali metal atom. The polarizability of Li@O_top_–Ca_12_O_12_ complex is 582 a.u. The polarizability of Na@O_top_–Ca_12_O_12_ is 662 a.u. which is higher than that of Li@O_top_–Ca_12_O_12_. While the polarizability of K@O_top_–Ca_12_O_12_ complex (1062 a.u.) is higher than that of Na@O_top_–Ca_12_O_12_. The results of polarizability show clear dependence on size of alkali metal atom. By the increase in size of alkali metal atom, its ionization energy decreases. Hence, the donation of electron becomes easier.^13^ The monotonic increase in polarizability seen for *exo*-complexes is not observed for endohedral complexes. For endohedral complexes, the polarizability of *endo*-Na@Ca_12_O_12_ is lower than those of *endo*-K@Ca_12_O_12_ and *endo*-Li@Ca_12_O_12._

This exception is also observed for hyperpolarizability where the hyperpolarizability of *endo*-Na@Ca_12_O_12_ (3.5 × 10^4^ a.u.) is lower than that of *endo*-Li@Ca_12_O_12_ (1.5 × 10^5^ a.u.) and *endo*-K@Ca_12_O_12_ (1.0 × 10^6^ a.u.). Overall, for these M@Ca_12_O_12_ complexes, the hyperpolarizability of most of the geometries ranges up to four powers of magnitude (10^4^). For exohedral doped systems, the hyperpolarizability shows a monotonic decrease where the hyperpolarizability of Li@O_top_–Ca_12_O_12_ is the highest in the series (2.4 × 10^4^ a.u.) whereas K@O_top_–Ca_12_O_12_ has the lowest hyperpolarizability (1.2 × 10^4^ a.u.). Such a decreasing trend for *β*_o_ can be attributed to the geometric distance between M and oxygen atom of the ring, rather than the ionization potential of alkali atom M. The vertical distance (between metal and atoms of ring) can dominate the trend of the *β*_o_ values, which is responsible for the decrease of *β*_o_ value in M@O_top_Ca_12_O_12_ (M = Li, Na, and K) series with increasing the alkali atomic number.^13^

Observing the hyperpolarizability for each of these series *i.e.* Li@Ca_12_O_12_, Na@Ca_12_O_12_ and K@Ca_12_O_12_ nanocages, it is evaluated that for Li@Ca_12_O_12_ series, the highest value of hyperpolarizability is calculated to be for the geometry with Li lying endohedrally *i.e.* 1.5 × 10^5^ a.u. while the other geometries with Li lying exohedrally show the hyperpolarizability of 2.4 × 10^4^ a.u. The same trend is seen for Na and K doped systems (endohedral complexes show higher hyperpolarizability than the exohedral complexes).

These results can be justified based on the results of VIP. The geometries with lower VIP have higher hyperpolarizability and *vice versa*. For example, the VIP of *endo*-Li@Ca_12_O_12_ (3.09 eV) is lower than Li@*exo*-@Ca_12_O_12_ (3.94 eV) while the hyperpolarizability of *endo*-Li@Ca_12_O_12_ (1.5 × 10^5^ a.u.) is higher than Li@*exo*-@Ca_12_O_12_ (2.4 × 10^4^ a.u.). Similarly, the VIP of *endo*-Na@Ca_12_O_12_ (3.24 eV) is lower than Na@*exo*-@Ca_12_O_12_ (3.92 eV) and that of *endo*-K@Ca_12_O_12_ (2.63 eV) is lower than K@*exo*-@Ca_12_O_12_ (3.57 eV). Both of these Na and K doped Ca_12_O_12_ complexes show the same trend for hyperpolarizability as that shown by Li@Ca_12_O_12_*i.e.* hyperpolarizability of *endo*-Na@Ca_12_O_12_ (3.5 × 10^4^ a.u.) is higher than Na@*exo*-@Ca_12_O_12_ (1.6 × 10^4^ a.u.) and that of *endo*-K@Ca_12_O_12_ (1.0 × 10^6^ a.u.) is higher than K@*exo*-@Ca_12_O_12_ (1.6 × 10^4^ a.u.). Moreover, the HOMO–LUMO gaps also justify the trend. The *endo* geometries with lower H–L gaps also show the higher hyperpolarizabilities when compared to their respective *exo*-geometries. The hyperpolarizability values of these clusters are also compared with the hyperpolarizability response of some well known NLO standards such as urea, *p*-nitroaniline and KDP. The hyperpolarizability values of urea, *p*-nitroaniline and KDP are 31.18, 76.76 and 376.75 a.u., respectively which are much lower than the hyperpolarizability values calculated for our systems where the values reach up to 1 × 10^6^ a.u.

### 3.4. Controlling factors of hyperpolarizability

Two level model is employed to understand the controlling factors of hyperpolarizability. According to two level model*β*_o_ ≈ Δ*μ* × *f*_o_/Δ*E*^3^where Δ*μ* is difference of dipole moment between the crucial excited state and the ground state, *f*_o_ is the oscillator strength and Δ*E* is the transition energy between the ground state and the crucial excited state. This relation shows that *β*_o_ is directly proportional to Δ*μ* and *f*_o_ while it is inversely proportional to Δ*E*^3^. As the relation shows that probabilities of electronic transitions are directly related to *f*_o_ and inversely related to Δ*E*, so the crucial excited state is chosen to be the one with larger *f*_o_.

The transition energies of the studied compounds are very small ranging from 0.63–1.29 eV. The hyperpolarizability values calculated for these compounds show an inverse relation with these Δ*E* values. *endo*-Li@Ca_12_O_12_ and *endo*-K@Ca_12_O_12_, show the hyperpolarizabilities of 1.5 × 10^5^ a.u. and 1.0 × 10^6^ a.u., respectively. These values are the largest of all the values (compared to the values calculated for all the other geometries of Li, Na and K@Ca_12_O_12_ nanocages). The transition energies for these two cases are the lowest of all *i.e*. 0.65 and 0.63 eV for *endo*-Li@Ca_12_O_12_ and *endo*-K@Ca_12_O_12_, respectively. This clearly reveals the inverse relation between *β*_o_ and Δ*E*. Moreover, for each of these series *i.e.* Li@Ca_12_O_12_, Na@Ca_12_O_12_ and K@Ca_12_O_12_, it is observed that the highest hyperpolarizability is calculated for the geometry which contains metal endohedrally. While it is also observed that for each metal, the exohedral complex has high transition energy than the corresponding endohedral complex (Table 2).

The oscillator strength (*f*_o_) which possesses the direct relation with hyperpolarizability (*β*_o_) is consistent with *β*_o_ for all complexes *i.e.* Li@Ca_12_O_12_, Na@Ca_12_O_12_ and K@Ca_12_O_12_. *endo*-Li@Ca_12_O_12_ shows the highest hyperpolarizability of 1.5 × 10^5^ a.u. and this geometry has the highest value of *f*_o_*i.e.* 0.21 as compared to the corresponding exohedral Li@O_top_–Ca_12_O_12_. The same is the case with Na and K doped Ca_12_O_12_ systems, both these systems possess highest hyperpolarizabilities of 3.5 × 10^4^ and 1.0 × 10^6^ a.u. for endohedrally lying Na and K geometries with the oscillator strengths of 0.18 and 0.22, respectively, higher than the *f*_o_ of corresponding exohedral geometries. Overall, out of all the three types of systems, the highest oscillator strength is 0.22 for *endo*-K@Ca_12_O_12_ which also has the hyperpolarizability of 1.0 × 10^6^ a.u. (highest of all the other configurations).

For Δ*μ* (difference of dipole moment between the crucial excited state and the ground state), which possesses direct relation with *β*_o_, it is observed that out of all *endo*-M@Ca_12_O_12_ (where M = Li, Na and K), the highest value for change in dipole moment is shown by *endo*-K@Ca_12_O_12_. Along with highest Δ*μ*, highest *β*_o_ is also shown by the same complex (Table 2). In a similar way, for *exo*-M@Ca_12_O_12_ (where M = Li, Na and K), the highest Δ*μ* is observed for *exo*-Na@Ca_12_O_12_ which also possesses the highest hyperpolarizability among these three, showing direct relation of Δ*μ* with *β*_o_.

## 4. Conclusions

Because of the diverse applications of NLO compounds in different fields, excess electrons containing NLO compounds using Ca_12_O_12_ nanocages have been designed. These alkali metals doped nanocages are thermodynamically stable with interaction energies up to −77.47 kcal mol^−1^. Along with thermal stability, these doped systems show excellent nonlinear optical responses when doped with alkali metals because alkali metals donate their electrons to the nanocages and thus introduce excess electron into them. The presence of excess electron has been confirmed through the NBO analysis, HOMO analysis and partial density of states (PDOS) spectra. Furthermore, the participation of excess electron for enhancing the NLO response of these nanocages has been confirmed through the hyperpolarizability of these doped nanocages, which is a decisive factor for the NLO response of compounds. The electrides possess remarkable nonlinear response where the highest hyperpolarizability can reach up to 1.0 × 10^6^ a.u. for *endo*-K@Ca_12_O_12_. This electride has the lowest Δ*E* of 0.63 eV among all compounds studied here. Moreover, the controlling factors of hyperpolarizability have been explored through TD-DFT calculations and two level model. The detailed study of these excess electron compounds marks them as capable of being used in NLO materials.

## Conflicts of interest

Authors declare no conflict of interest.

## Supplementary Material
